# Review of the Proceedings of the American Society of Dental Surgeons

**Published:** 1848-01

**Authors:** E. Taylor


### REVIEW OF THE PROCEEDINGS

### *Of the American Society of Dental Surgeons, for* 1847,

BY E. TAYLOR, D. D. S.

This Society held its eighth annual meeting at Saratoga Springs, commencing Aug. 3d, 1847. The report of its proceedings we find in full in the American Journal of Dental Science for October. The proceedings occupy about nine pages. This, 'tis thought, is more room than we can well spare in the Register. And yet, the doings of an institution having the same objects in view as our own Society, should receive our attention.

We are gratified to have the assistance of even a single individual in promoting the interests and elevating the standard of our profession. How much more should we rejoice, to have the aid and assistance of this Society, embracing so many of the most talented and influential members of our profession.

About thirty members appear to have been in attendance. The attention of the Society was almost wholly occupied with the "amalgam controversy." This subject appears to be a most hydra-headed monster. It has come up, we believe, in some form, at every meeting of the society since its organization, and we had thought it killt and dead every time; but the ugly thing won't stay dead, but rather seems to send forth a new progeny from every defeat more troublesome than the former. The capital appears now to be taken, and the Guerillas dispersed; shall we not now look for submission and peace? We hope so; and as the smoke and noise of the conquest is past, we trust that we shall not be considered as giving "aid and comfort" to the enemy, by a calm review of the manner in which the war has been conducted. We admit there has been just cause for a declaration of war. Barbarians had invaded the realms of true science, and by pillage and desolation, (allow a little for metaphor), were laying waste some of the fairest portion of our heritage; and it was important that something should be done to stay their progress. The society was organized for the express purpose of guarding, and promoting the interests of true science, and it was therefore evidently its duty to engage in the conflict. The war must, however, be waged in accordance with the enlightened spirit of the age, particularly by the party in defence of the interests of light and truth. No bloodhounds must lead on the attack. Private property must be respected, peaceful citizens who are giving no aid and comfort to the enemy, must not be disturbed; and utter extermination, without regard to age or sex, must not mark the progress; nor must the captives be hung up without the benefit of trial or clergy. The moral sense of mankind must not be outraged by a departure from those principles which govern human society. Has the society then conducted it in a manner to meet the just expectations of the friends of science? Let us examine. In the first place, the society declares it to be malpractice to use amalgams in filling teeth, and the constitution declares that any member may be expelled for misconduct, malpractice in business, or other sufficient cause. It is therefore clear that the society has the power to determine what is malpractice, and we think their action was evidently proper in the case. The next step was to instruct the Recording Secretary "to give written notice to every member of this society, charged by another member with plugging teeth with *mineral paste* of *any* kind, that the society has pronounced the use of that class of articles for this purpose, *malpractice*, and such member, thus charged, that this society will act upon his case at its next meeting." This is commendable, as it is in accordance with the principles of jurisprudence to notify the accused of the charges preferred, and the time and place of investigation, where he may meet his accuser face to face, and plead his own cause. At the next meeting the subject occupied so much attention that we cannot give more than a summary.

A committee of investigation was appointed, to call on each of the members then in the city, (New York,) and ascertain from each member whether he had used any amalgam in the course of his practice, especially during the last year, or approved of its use. The committee called upon twenty-one resident and twenty-one non-resident members. The non-resident members unanimously disapproved of its use, and did not use it; ten residents also disapproved of, and did not use it; five others were willing to pledge themselves not to use it, and the other six were unwilling to discontinue its use. The report was referred to a committee of five, to report some plan of action for the society to take. The committee reported "that they had deliberated carefully upon the matter referred to them, and that their unanimous opinion is that any amalgam is not only unfit, but dangerous when used for the purpose of filling carious teeth or their fangs. That it is therefore the imperative duty of the society to express most distinctly, and unqualifiedly, their disapproval of its use for the above named purposes under any circumstances—and submit the following resolutions:

*Resolved*, That the American Society of D. S., under the conviction that any amalgam, when used under *any* name, is not only unfit, but dangerous, when used for filling the teeth or their fangs, do hereby pronounce the use of amalgams as malpractice.

And furthermore, *Resolved*, That any member of this society who shall hereafter refuse to sign a certificate, pledging himself not to use any amalgam, and, moreover, protesting against its use under any circumstances in Dental practice, shall be expelled from this society."

The resolutions were adopted. With a view, however, to make the matter more definite, and also that equal and exact justice might be done to all, the following resolutions were unanimously adopted:

*Resolved,* That the Recording Secretary be, and is hereby directed to forward to every member of this society, within thirty days after the 10th of August, 1847, a printed copy of the resolutions passed at the convention of the A. S. of D. S., relative to the use of amalgams under any name or form.

*Resolved,* further, that the Recording Secretary be requested at the same time, to transmit to the several members as aforesaid, a printed protest or certificate in accordance with the above resolutions, notifying each member that unless he shall subscribe with his own proper signature, the said protest or certificate, and return the same to the Recording Secretary within sixty days from the time the said notice is issued, or in the case of their living out of the United States, within one year from the first day of August, 1847, he shall be considered ineligible to be a member of the A. S. of D. S., and shall stand expelled from the same.

And it is furthermore *Resolved,* That the Recording Secretary be, and he is hereby directed without further reference, to strike from the roll of this society, after the expiration of the above specified terms, the names of such members as may have failed to comply with the terms and conditions set forth in the foregoing resolutions."

In accordance with which, the Recording Secretary issued, and directed to each member, the following blank protest or certificate:

"I, ———, hereby certify it to be my opinion and firm conviction that any amalgam whatever, whether used under the name of mineral paste, adamantine cement, succedaneum, diamond cement, Chinese cement, lithodeum, albaster cement, or any other way designated, is not only unfit but dangerous when used for plugging teeth or their fangs.

And I pledge myself never, under any circumstances, to make use of it in my practice as a Dental Surgeon.

And furthermore, as a member of the A. S. of D. S., I do subscribe and unite with them in their protest against the use of the same.

Given under my hand and seal this, — day of ——, 184–."

Well, if this is not the most perfect farce, upon an attempt to proceed according to law and order, that we have ever met with, we will "give it up." Every man is guilty until he proves or swears himself innocent, and that within thirty days, whether sick or well. "It is his business to be well," at home or abroad. "Pshaw, what business has a Dentist to be abroad?" Mail failures or no. "A professional man ought to know his duties by intuition." But perhaps he would like to say a word or two by way of explanation. "Mr. Secretary, cut him off." But stop—stop—I'll— "Your'e too late." The guillotine has fallen and away rolls the headless trunk.

But as the "*sober* second thought" of the society, at its next annual meeting, rescinded this action, it is not worth while to dwell upon it.

Feeling jealous of the reputation held by the medical profession of killing scientifically, they determined to have the constitution on their side, and amended the sixth article to read as follows:

"Any member or members of this society may be expelled from this body for immorral conduct, malpractice in business as declared to be such by this society, or for refusing to comply with the requirements of this constitution and by-laws, or the requirements of this society, or other sufficient cause, on the motion of any member, seconded by another, at any regular annual session, in which case a majority of two-thirds of the members present shall be required."

A passing notice of this article, to show the inconsiderate haste with which many of their proceedings have lately been marked. We hope the reader will scan the article over. "Any member or members," retail or wholesale, "may be expelled from this body"—pray, for what other body are they legislating—"for immoral conduct, malpractice in business, as declared to be such by this society, (it makes no difference how gross or palpable the malpractice may have been, unless it had been declared so by the society, the member may practice it with impunity; unless an ex-post facto law should be made to reach him,) or for refusing to comply with the requirements of this constitution and by-laws,

6

(which of this by-laws,) or the requirements of this society," &c. Well, surely they are outflanked now. There is latitude enough of power for an inquisition, and the society may require of each member to report every thing they may do, say or think; and verily, they don't come far from such a requisition, at least professionally.

But to return to the war. The society, with this amplitude of power, buckled on the armor again for the conflict, and

"*Resolved*, That those members whose names appear and constitute the third and fourth classes of the Recording Secretary's report, (viz: those who did not respond to the circular, and those who refused to sign the protest) be, and are hereby *required* to sign and return to the said Secretary the following protest." The protest is the same as the former, except that the word "dangerous" is omitted in the certificate.

The Secretary was directed to "forward the resolutions and protest to each of said members, and respectfully request them promptly to respond, either by signing the protest or declining to do so, that the society may be able to act more advisedly upon the case of each one who does not signify his desire to withdraw from the society, previous to the next regular annual session." And the Secretary was directed to report to the annual session of 1847, "that the case of each one might be acted upon by the society at that meeting, in accordance with the testimony *thus* furnished."

Truly this looks like the "dog returning to his vomit, and the sow that was washed to her wallowing in the mire." After their former defeat we had hoped they would have changed their tactics, and return to the wisdom with which the campaign was opened.

If the society is constituted of members who are only to be restrained from malpractice by a solemn and formal pledge, we had much mistaken its character, and the sooner it disbands the better; for if such ethics obtains, we fear its existence will be of little moment in advancing the character of the profession. What would any one say of the discipline of a church that would require of its members a solemn protest and pledge not to get drunk? and must the members of a learned profession, banded together for the purpose of elevating the character of their profession, proclaim to the world that they have not confidence enough in the honor and character of even the select few, without a formal certificate, pledge and protest duly signed and sealed, and filed away.

"But the sound of the bugle calls to arms," so we must away and return to the place whence we started, viz: the proceedings of the last annual meeting. The cases of the different individual delinquents were taken up. That of Dr. J. Lovejoy was the first definitely acted upon. The accused acknowledged he used the amalgam, and intended to continue its use. It was therefore resolved and carried by a two-third vote, "That Dr. John Lovejoy be, and he is hereby expelled from the American Society of Dental Surgeons for refusing to comply with their positive mandates, in *using*, and refusing to discontinue the use of amalgams for filling teeth."

This resolution was marked A. for Axe we suppose, and on this resolution some three others were expelled. Against the action of this resolution on all the delinquents present to defend themselves we take no exception, save that we should have omitted all between the words "surgeons" and "amalgams." except "for using."

But as disobedience to orders is a more heinous offence than malpractice, we suppose it was best to decapitate them under this charge.

The case of D. Morrow was taken up. It does not appear whether he was present or not; but he was expelled on the follow resolution, which was marked B. we suppose for Bowie-knife or Broadaxe:

"*Resolved*, That D. Morrow be, and he is hereby expelled from the A. S. of D. S., for refusing to sign their positive mandate, (terrible) by refusing to sign the protest of this body, relative to the use of amalgams for filling teeth." Some five others, including Dr. E. Baker, the gallant general of the opposing forces, fell under this resolution, and although we cannot approve his cause, yet we cannot but admire the generalship displayed in conducting his cause. But his forces had finally been discomfitted, and as a prisoner of war he was led forth to his trial. The victorious general, in deference to his great talents, modestly declined appearing in triumph over his fallen foe, and with his aidecamp retired, and the solemn ceremony of law commenced. The prisoner at the bar was called upon to say why sentence of death should not be passed upon him. "He responded to the effect, that the action of the society on this subject had been entirely unconstitutional. He then expressed his dissatisfaction with the society, for having expelled members while those were absent who took an interest in their proceedings, and who would have been glad to have defended the accused. He then took up the general subject of amalgams, and spoke in its defence," but his plea was not sustained, and he was condemned to die, but was allowed to choose between the Axe and Bowie knife. He chose the latter, and calmly and heroically received the polished shaft to his vitals, and the court adjourned. The case of Dr. C. C. Allen was taken up, but after considerable halting and wincing, he finally submitted.

In view of good and sufficient evidence, to the effect that Dr. C. Starr Brewster had been in the habit of using amalgams for filling teeth, and that it was his intention to continue said practice, it was

*Therefore Resolved*, That he be no longer considered an honorary member of the A. S of D. S.

J. Smith Dodge's case was then taken up, and J. B. Rich, chairman of the committee formerly appointed to investigate certain charges, reported, that he had summoned Dr. Dodge, and that he had not paid any attention to it. Whereupon he was expelled for *contumacy.* (*Rich.*)

The case of Dr. N. C. Keep was taken up. He was in favor of a treaty of peace, based upon mutual compromise; but the society was as inexorable as death, and he consequently resigned. The cases of several others were laid over for another year, and they left to suffer the gloomy forbodings, more fearful than death itself.

The committee on the case of Dr. J. Allen, relating to the course pursued by him towards the society, in respect to an invention which he has had patented, for remedying deformities of the face, reported,

"That, whereas, after having freely offered the benefits of his invention to every member of this society, to be used gratuitously, that without their knowledge he had secured a patent therefor; and that whereas he saw fit, at a subsequent period, to make an attempt to exact from members who might be disposed to use it, a certain per centage for the privilege of using it, your committee deem his conduct highly reprehensible.

And whereas, Dr. Allen has made such use of the fact of a gold medal having been presented to him by the society, as the society do not contemplate in making such awards, and cannot approve or sanction; therefore,

*Resolved*, That the censure of this society be passed upon Dr. Allen for having acted in a manner derogatory to his character as a professional gentleman, and attended with consequences highly detrimental to the society of which he is a member."

We have heard of such a thing out here in the west as "Snap Judgment," but we believe this is one of the first cases that we have met with.

Now, we can imagine old Mrs. Partington taking off her spectacles at the above resolutions and whereas, and in holy horror looking up and exclaiming, "Awful! Well, surely if he had escaped the fury of the storm, yet vengeance will not suffer him to live." But we are happy to say that the Dr. is still alive, aye, and kicking too, and although it is not for us to say that he has "shook off the venomous reptile into the fire, and felt no harm," yet we would remark that he has issued a "Refutation and Vindication" in pamphlet form, in which he shows at least that there is another side to the question. The Dr. states in his pamphlet, that he had secured his patent *previous* to the meeting of the society in 1845; and that this fact was known to several leading members of the society, as he did not hesitate to speak of it freely; and that he acted in accordance with the counsel of those men, whose names he may give hereafter if it becomes necessary. He also contends that as his invention was not designed to mitigate the sufferings of humanity, it should come under a different head altogether in considering the propriety of a patent. He also denies having offered its free use to the members, but only submitted it to the consideration of the society, and was disposed to grant the members special privileges, and that he considers those privileges best protected by letters patent, and the requirement of a per centage of the profits arising from its use. In reference to the third whereas, the Dr remarks: "If they think that I have given it too much *publicity*, I will state most *positively* I have never written three lines on the subject since I received it. I have shown it to some of my friends who desired to see it, for I did not know that it was wrong to do so, and some of my editorial friends have given it a passing notice in their papers, from which I have taken a few *extracts*, which may be seen upon my circular."

We do not stand forth in the Dr.'s defence, but have thought it proper to give a synopsis of his own defence. Our views on the propriety of patenting any thing connected with the profession have been freely expressed before our society of the west, and do not require to be repeated here; but we most heartily concur with him in the following remarks on the propriety of the society's action:

"And now I would ask, if the course pursued towards me in this case, can be regarded as a safe and judicious one. I think not, for by this precedent any member, through personal envy, or private pique, may be hurled unceremoniously from his station, however exalted. Though he may have been struggling for twenty years, to attain the highest degree of professional fame, in his absence from a meeting of the society, FALSE charges may be preferred against him, his purest and best motives may be impugned, and a construction put upon them that he never intended. One side of the question only is heard, for he is denied a hearing; no defence or explanation is admitted; he is arraigned, tried and condemned, and his sentence of condemnation at once published to the world, and the first intelligence he has of it, meets his eye in the public journals of the day."

It will be recollected that the award was made to Dr. Allen in 1845; he attended the meeting of the society in 1846, and not only were no charges preferred against him, but he was elected one of their Vice Presidents. But at the meeting in 1847 the above action was had on his case. We are very much reminded of Capt. Tobin's anecdote of his fellow-countryman, inattentively engaged in sucking raw eggs, when one, a little older than the others, chirped as it went down; he very gravely replied, "By the powers, my friend, you spoke too late."

Resolutions were passed, making it the duty of members to report any improvement in the management of disease, the condition of the profession, and novel results in practice. And others, declaring it derogatory to retain patentees, or any who hold professional secrets, as members.

The following were elected officers for the ensuing year: E. Parmly, Pres't.; L. Roper, E. Noyes, W. H. Goddard, Vice Pres'ts.; J. B. Rich, Cor. Sec.; A. Westcott, Rec. Sec.; J. H Foster, Librarian; C. A. Harris, A. Westcott, Wm. H. Dwindle, Editors. Executive and Examining Committee, Dr. J. H. Foster. J. B. Rich, E. J. Dunning, E. Parmly, J. M'Ilhany, C. A. Harris, H. N. Tenn.

The Society adjourned to meet at Saratoga Springs, on the first Tuesday in August, in 1848. We are sorry that they did not hold to their first purpose to meet at Niagara Falls, as we fear that Saratoga water does not agree with them. It is evidently too gasseous. We should have expected a much more deliberate and wise course of proceedings "'mid Niagara's thundering roar," and as we hope to visit the lake next summer, we might have had the pleasure of looking on.

